# Eccentric Occlusal Loads Remodel the Dentoalveolar and Temporomandibular Biomechanical Unit in Rats

**DOI:** 10.1111/joor.70284

**Published:** 2026-08-17

**Authors:** Haochen Ci, Umama Ali, Zhiyuan Yang, Ayush Bakhandi, Nhu Huynh, Christine Nguyen, Brandon Lee, Youngho Seo, Robert Flavell, Alireza Hatamifar, Rishi J. Gupta, Rebeka Silva, Yongmei Wang, Bo Wang, Sunita P. Ho

**Affiliations:** ^1^ Department of Preventive and Restorative Dental Sciences School of Dentistry, University of California, San Francisco San Francisco California USA; ^2^ School of Mechanics and Aerospace Engineering, Dalian University of Technology Dalian People's Republic of China; ^3^ Department of Radiology and Biomedical Imaging School of Medicine, University of California, San Francisco San Francisco California USA; ^4^ School of Dentistry, University of Washington Seattle WA USA; ^5^ Neuroscience Graduate Program, Department of Pharmacology University of California Davis California USA; ^6^ Department of Nuclear Engineering University of California Berkeley California USA; ^7^ UCSF Helen Diller Family Comprehensive Cancer Center San Francisco USA; ^8^ Department of Pharmaceutical Chemistry University of California San Francisco California USA; ^9^ Department of Oral and Maxillofacial Surgery University of California, San Francisco San Francisco California USA; ^10^ Department of Oral and Maxillofacial Surgery VA Medical Center San Francisco California USA

**Keywords:** biomechanics, dentoalveolar joint, masticatory complex, occlusal loads, temporomandibular joint

## Abstract

**Background:**

Altered occlusal mechanics are critical regulators of the structure and function of the dentoalveolar and temporomandibular joints (DAJ, TMJ); however, the multiscale biological and mechanical consequences of sustained occlusal rise on the TMJ are not understood.

**Objective:**

This study establishes a proof‐of‐concept for using an occlusal rise (OR) to investigate how the resulting chronic eccentric loading influences the DAJ and TMJ across mechanical, structural, and metabolic domains.

**Methods:**

Rats were subjected to increased occlusal forces by placing a 0.5 mm metal wire and dental composite on the maxillary molars, producing a total occlusal rise of 1–1.5 mm. The DAJ and TMJ condylar bones in OR (*N* = 7) and control (*N* = 3) groups were evaluated using in vivo micro‐CT at days 1, 14, and 35. One rat/group underwent ^18^F‐NaF PET, complemented by autoradiography and histology. Finite element modelling (FEM) based on CT‐derived geometries was used to evaluate TMJ reaction forces and stress under OR‐loads.

**Results:**

Widened PDL space and interradicular bone remodelling were accompanied by reduced BMD with relatively preserved BV/TV, indicating redistribution rather than uniform loss of mineralized tissue in the DAJ. Progressive changes in condylar physical properties, with deviations between anatomical and mechanical axes, reflected adaptive load transmission. Increased ^18^F‐NaF in TMJ indicated elevated metabolic activity. Histology confirmed ectopic cartilage‐like tissue formation. FEM demonstrated buffering of TMJ stresses followed by temporal structural reorganisation.

**Conclusions:**

Sustained OR drives coordinated, time‐dependent remodelling across the DAJ‐TMJ biomechanical unit through coupled mechanobiological adaptation, providing a mechanistic framework linking eccentric occlusal loading to TMJ remodelling and pathophysiology.

## Graphical abstract

Eccentric occlusal loads remodel the dentoalveolar and temporomandibular joints in the masticatory biomechanical unit in rats. (A) Bite block as an occlusal rise (OR) on the rat maxillary first molar (green region) is shown. (B) ^18^F‐NaF Positron emission tomography localised regions of higher metabolic activity (higher uptake in the left condyle compared to the right condyle). (C) Finite element modelling (FEM) on CT‐derived geometries evaluated TMJ reaction forces and stress under OR‐loads and demonstrated buffering of TMJ stresses followed by temporal structural reorganisation.

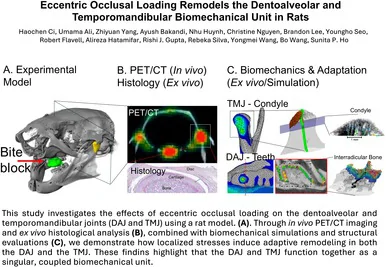

## 1 Introduction

Chewing loads, modulated by food hardness, are central to shaping and maintaining the masticatory system [1, 2, 3, 4, 5]. Efficient load transfer requires coordinated biomechanical coupling across multiple soft and hard tissues such as the periodontal ligament (PDL) and alveolar bone of the dentoalveolar joint (DAJ), and the cartilage and trabecular bone of the temporomandibular joint (TMJ). The collective function of the many soft and hard tissues of the fibrous joint, the DAJ, and the diarthrodial joint, the TMJ, is essential for mastication [6]. However, most research focuses on either the individual tissues of the DAJ or the TMJ, thereby missing emergent responses that arise from the system‐level coupling. As a result, the mechanobiological adaptation of the DAJ‐TMJ unit under sustained occlusal loads remains poorly understood, even as evidence linking altered occlusal forces to TMJ dysfunction continues to grow [7, 8, 9, 10]. Addressing this gap is essential for elucidating the mechanistic underpinnings of occlusion‐related TMJ disorders and developing targeted interventions.

Animal studies have demonstrated that elevated occlusal loading, achieved through alterations in vertical dimension, drives structural and temporal remodelling within the DAJ and/or the TMJ [9]. Unilateral occlusal perturbations alter bone remodelling in the unloaded condyle and induce histological remodelling in the contralateral joint capsule [11, 12, 13]. These findings highlight asymmetric loading as a potent mechanobiological cue capable of triggering changes in condylar morphology and necessitating compensatory adaptation [9, 11, 14, 15]. Persistent perturbation, over time, accelerates cartilage and subchondral bone remodelling by significantly altering the condylar cartilage thickness and bone volume fraction [16]. In growing rats, vertical dimension changes mandibular deviation and asymmetric condylar development, underscoring its spatiotemporal impact on joint symmetry [17, 18]. Furthermore, the maintenance of condylar morphology depends on masticatory loading, as evidenced by trabecular thickening and cartilage proliferation under altered force environments [16, 17, 18]. Despite these insights, the explicit role of DAJ‐driven perturbation (occlusal rise/hyperocclusion/biteblock) in shaping TMJ structure remains underexplored.

Building upon this foundation, this proof‐of‐concept study investigates how the protracted effect of a unilateral occlusal rise (OR) can remodel the DAJ–TMJ complex by longitudinally tracking key structural parameters. In this longitudinal study, we mapped the evolving effects of unilateral occlusal load in rats on the DAJ and TMJ and then applied finite element modelling (FEM) to adapted anatomy to compute stresses and strains within alveolar and condylar bones. By integrating physical measures such as the temporal changes in PDL space, moment of inertia of alveolar and condylar bones, condylar morphology, and bone density, this study delineates a biomechanical continuum linking the DAJ and TMJ. In doing so, it provides a systems‐level perspective on how occlusal perturbations propagate across load‐bearing joints, offering insight into the mechanobiological basis of occlusion‐related TMJ pathology.

## 2 Materials and Methods

### 2.1 Animal Model and Specimens

All experimental protocols followed the guidelines of the Institutional Animal Care and Use Committee (IACUC), and experiments were conducted as per the animal protocol AN200023. A 0.5 mm metal wire and dental composite with a total occlusal rise of 1–1.5 mm was generated on the left 1st maxillary molar of Sprague–Dawley male rats (*N* = 7) at age 12 weeks [19], while no procedure was performed on control rats (*N* = 3) (see Supporting Information for statistical sample size). The experimental rats were checked every day to ensure the OR was intact (Figure S1). Rats were euthanised at the end of approximately 5 weeks (~35 days) at 17 weeks of age. All rats in both groups (control, OR) were given unlimited access to water and were fed hard food for the duration of the study. The rats were weighed daily (Figure S1). Outcome measures from rats in both groups are shown in Figure 1A.

**FIGURE 1:**
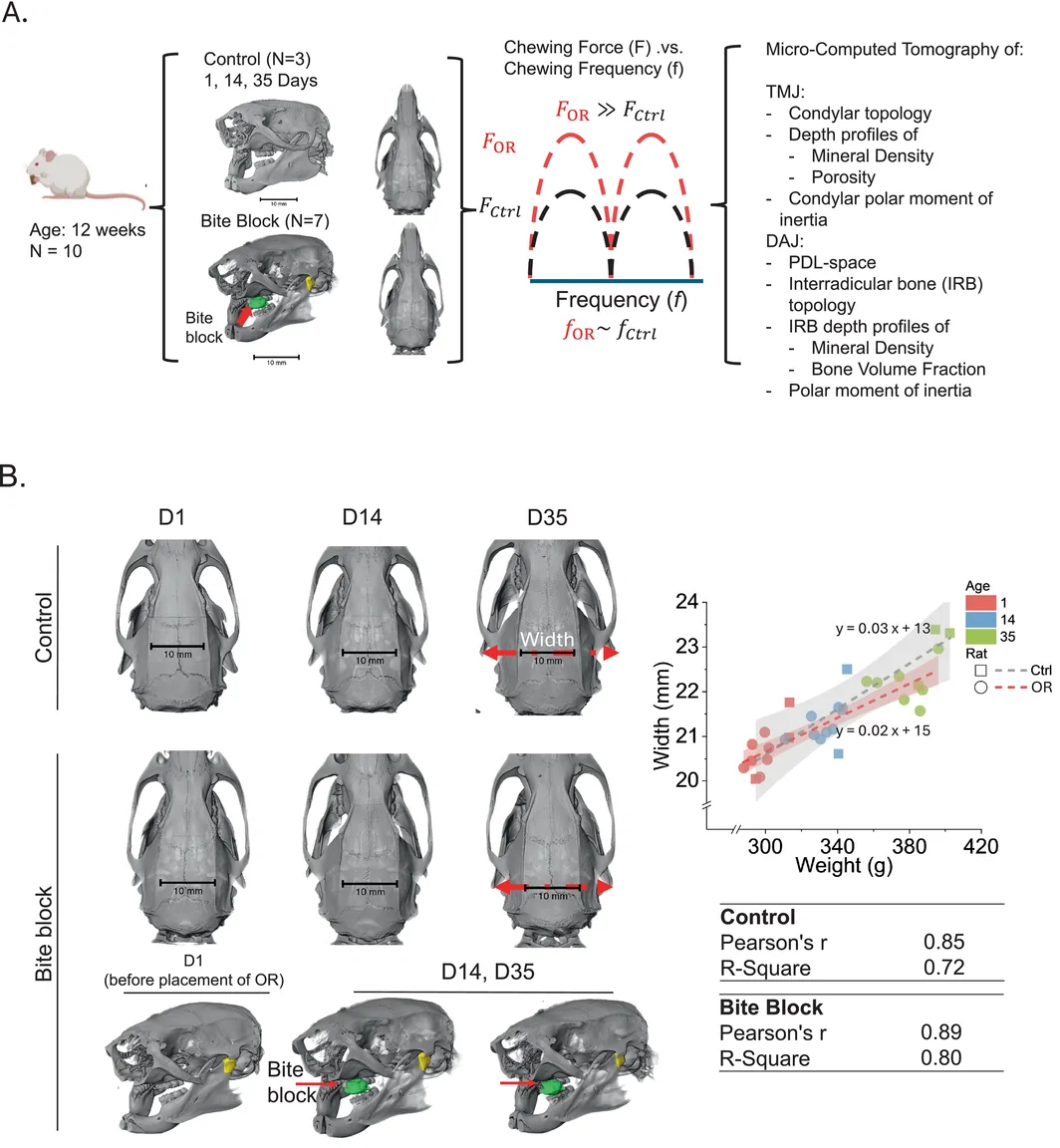
Experimental framework and temporal outcomes of a rat occlusal rise model. (A) Experimental Schematic: Illustration of experimental design used to assess the effects of occlusal rise (OR) (*N* = 7) compared with a regular diet (control, hard food, *N* = 3) in rats. Chewing force magnitude (F) and frequency (f) across the two groups are illustrated. The magnitude of force by OR is greater than that of hard food only because of the pre‐collision before chewing. The mastication frequency is assumed to be similar because of the same food hardness in the OR and control groups. Spatiotemporal (1, 14, and 35 days) morphological changes in the condyles were measured using X‐ray micro‐computed tomography (X‐ray CT). (B) Temporal differences in head shape and size ‐ OR rats trended toward smaller width between condyles compared to controls: Temporal changes in skull morphology between control (grey) and OR (red) rats are shown across three time points: Day 1 (D1), Day 14 (D14), and Day 35 (D35). Green regions show the OR, and the yellow regions show condyles.

### 2.2 X‐Ray CT Imaging

X‐ray CT imaging of all 12 rats (7 from OR, 3 from Control) at an energy of 50 kVp was performed at time points: Day 1 (D1), Day 14 (D14), and Day 35 (D35) at a spatial resolution of 10 μm. The digital volumes were used to evaluate bone volume fraction (BVF, bone volume/(total volume = pore volume + bone volume)) and morphology of the condyle and topology of the condylar surface. The bone mineral densities (BMD) in mg/cm^3^ of left and right condyles from both groups were evaluated (Figure S2). Associations between BMD and BVF depth profiles from the condylar surface into bone within and across both groups were mapped.

#### 2.2.1 Morphology of the Periodontal Complex and Condylar Bone

Interradicular bones of the first maxillary molars of the OR group were processed using AVIZO software (AVIZO 2024.1, Thermo Fisher Scientific, Waltham, MA, USA) and MATLAB (MATLAB R2024b, The MathWorks Inc., Natick, MA, USA). Evaluation of the periodontal ligament space (PDL‐space, mm), BMD, BVF, centroids and polar moment of inertia (PMI) of the OR‐ and control‐IRB and respective condylar bones at all three time points were estimated (Figures 3, S2, and S3). The surface distance between the alveolar socket surface and tooth roots was measured as the PDL‐space. The peak values and full‐widths at half maximum of the Gaussian fits to the data were used as the estimates of the PDL‐space at each time point for both groups. BMD was estimated by normalising the Hounsfield (HU) values of each voxel to the mineral density calibration curve (Figure S2). BVF was assessed as the bone volume divided by the total volume, which is the sum of bone volume and pore volume in any given region. Bone surface roughness (BSR) was determined separately for the mesial and distal side up to 4 mm of depth from the tooth‐IRB surface region, with the formula $H=\frac{1}{N}\sum_{i=1}^{N}\;z_{i}$ where z_i_ is the distance from the alveolar bone surface to 4 mm into bone, N is the number of points at which the distance was measured, and H is the mean value of z_i_ over all points. PMI with respect to the centroid was assessed from the coronal to apical side of IRB or from the condylar surface into the condylar bone by

$$I_{p}=\sum \left(dx^{2}+dy^{2}\right)\cdot f\left(x,y\right)$$

from binary images of the original CT greyscale images (Figure S2). Sagittal planes of the condyles were used to identify central sections to which parametric curves were fitted to determine the radius of curvature of the condyle. Porosity was evaluated in Avizo software, and results were analysed in MATLAB.

### 2.3 Correlative Positron Emission Tomography ( ^18^F‐NaF PET) and X‐Ray Computed Tomography (X‐Ray CT), and Histology

#### 2.3.1 Tracer Preparation

^18^F‐NaF was produced at the Radiopharmaceutical Facility of the University of California, San Francisco using the GEPETtrace cyclotron. Enriched ^18^O‐water was irradiated via the ^18^O (p,n)^18^F reaction to generate ^18^F fluoride ions. The fluoride ion ^18^F was trapped on a Light Sep‐Pak QMA cartridge preconditioned with 1 M Na_2_CO_3_ (5 mL) and H_2_O (10 mL). The trapped ^18^F fluoride was eluted from the QMA cartridge with an appropriate amount of 0.9% NaCl solution, ^18^F‐NaF, and prepared for injection.

#### 2.3.2 μPET/CT Acquisition and Data Analysis

To establish if a change in TMJ metabolism occurs under closed and open mouth conditions, a single Sprague–Dawley rat at 8 weeks was tracked at two time points ‐ 15 and 35 weeks following the OR procedure using μ‐PET/CT imaging (nanoScan μPET/CT, Mediso, Budapest, Hungary) in vivo. Both open and closed mouth conditions were performed on 35‐week‐old rats in vivo. The rat was anaesthetised with 2%–3% isoflurane in a mixture of medical‐grade oxygen gas, followed by intravenous tail vein injection of 18.5–25.9 MBq of ^18^F‐NaF. Co‐registered CT data were used for attenuation and scatter corrections. The vendor‐provided ordered subsets expectation maximisation (OSEM) algorithm was used for PET reconstruction.

All data analyses were performed by loading the PET and CT data into AVIZO software. To localise PET signal originating from non‐osseous tissues, CT images were used to identify PET‐positive bone regions, which were subsequently subtracted from the PET dataset, thereby isolating signal arising from adjacent softer tissues.

All PET imaging data were analysed using open‐source AMIDE software. Regions of interest (ROIs) were manually drawn over bones of interest on CT data and propagated to co‐registered PET. Standardised uptake values (SUVs) were calculated, normalised to the injected activity and the animal's body weight. Data were plotted using GraphPad Prism version 10.4.1 (GraphPad Software Inc.).

#### 2.3.3 Histology and Autoradiography

Histology was performed on the fixed (10% NBF), and decalcified (20% EDTA) 7‐μm‐thick sections of TMJ condyles (*N* = 1 from each group). Tissue sections were deparaffinised using standard protocols and stained with Haematoxylin and Eosin (H&E) and Safranin‐O (Saf‐O) and imaged using a light microscope (AXIO‐Z3, Zeiss Microscopy, Pleasanton, CA) [20]. Serial sections were used for autoradiography of ^18^F‐NaF. Sections were incubated with 1.85–5.55 MBq (50–150 μCi) of ^18^F‐NaF applied directly to the tissue for 1 h at room temperature. Following incubation, slides were washed three times in PBS, air‐dried, and affixed to a positron‐sensitive gadolinium oxysulfide (GOS) disposable scintillator for imaging with digital autoradiography over 24 h (iQID: QScint Imaging Solutions LLC). Relative‐intensity images were reconstructed from list‐mode data using a previously published open‐source Python‐based analysis toolkit [21].

### 2.4 Finite Element Analysis of the DAJ‐TMJ Unit

To investigate the biomechanical effects of OR on the DAJ‐TMJ biomechanical unit [22], finite element (FE) simulations were conducted (Figure S5). Bone and teeth were assumed as one singular unit. CT‐reconstructed images of the mandibular jaw with the teeth were processed in AVIZO to generate volumetric meshes, followed by manual correction of distorted or excessively deformed elements. The mesh type used was a 4‐node linear tetrahedron element (C3D4). Boundary conditions were applied by constraining the ligamentous attachment of the condyle to simulate joint reaction forces during occlusion. Properties used were 15 000 MPa for elastic modulus and 0.3 for Poisson's ratio for the singular unit [23]. Occlusal loads were applied to the occlusal surface of the first molar, and masticatory muscle forces were introduced at the insertion site of the masseter [22]. In the OR group, all occlusal and muscular loads were set at 1.5 times those of the control group to replicate excessive functional stimulation (Figure 5A). The resulting models were imported into Abaqus (Abaqus 2024, Dassault Systèmes Simulia Corp., Johnston, RI, USA) for finite element analysis to compare mandibular stress distributions between control and OR groups in rats.

The posterior margin of the TMJ was constrained to simulate a stiff ligament behaviour. Figure 5A, a1 illustrates free‐body diagrams of mandibular loading conditions for both control and OR groups. One unit of normal occlusal force was applied to the first molar in the control group. In the OR group, occlusal and muscular forces were increased by a factor of 1.5 (approximation) compared to the control group to represent the augmented mechanical loading conditions provided by the OR. A vertical upward force ten times the occlusal force was applied at the insertion point of the deep masseter muscle to simulate muscle contraction. A higher masseter muscle force was chosen as the muscle should contract to close the jaw and during unilateral chewing. It should be noted that alterations to the masticatory function through our OR model could change the masticatory muscles and their contractile capacity because of functional alterations brought about by changing the occlusal loads. The simulations were run on 3D CT reconstructed volumes from both control and experimental groups adapted over time (at all time points). Stress values at the constrained nodes of the TMJ and TMJ reaction force at time points D1, D15, and D35 were extracted for statistical analysis.

### 2.5 Statistical and Principal Component Analysis

Student's *t*‐test (unpaired, two‐tailed) was applied to all outcome parameters, and a *p*‐value < 0.1 (90% confidence interval) was used to identify trends toward significance between two different groups. A *p*‐value less than 0.1 was chosen as it is a proof‐of‐concept study with a smaller number of animals while accounting for larger intrinsic biological variability.

## 3 Results

Morphological features of DAJs and TMJs were estimated and compared among OR and control groups. Results from statistical analysis trended toward significant differences in these morphological properties between groups.

### 3.1 OR Rats Trended Toward Smaller Width Between the Condyles Compared to Controls

Temporal differences in width between condyles and skull morphology between control and OR groups are shown over time (1, 14, and 35 days). 3D reconstructions illustrated changes in width between condyles (red arrows). The scatter plot illustrates the correlation between the width of the condyles and body weight across different time points, with linear regression trends and statistical values reported for each group. Over time, the width of the condyles in the OR group diverged from the control group. The control rats exhibited higher skull growth rates (0.03 ± 13) and rat body weights (grey trend line) compared to the OR group (0.02 ± 15) (red trend line) (Figure 1B).

### 3.2 Widened PDL‐Space, Increased Interradicular Bone Roughness, Decreased BMD, and Increased BVF Trends in Rats With Occlusal Rise Compared to Controls

Figure 2A(i) presents the fitted trajectories of peak positions in the frequency distribution of the PDL‐space over time. In the control group, the peak position remained relatively constant around 0.14 mm from day 1 to day 35, with a near‐zero slope in the fitted curve (y = −0.00024·x + 0.14), indicating structural stability of the PDL and the absence of significant spatial drift. In contrast, the OR group (depicted in red) exhibited a marked rightward shift over time, with a positive slope in the fitted regression line (y = 0.0010·x + 0.13) (Figure 2A(iii)), reflecting a progressive increase in peak PDL‐space. Together, these results suggest that under sustained excessive and uneven occlusal loading, the PDL in the OR group underwent continuous spatial stretch and compression, driven by tooth‐bone interaction as related to occlusal loads. This trend indicates a progressive structural adaptation of the PDL in response to altered functional demands, potentially compromising its role in stress buffering.

**FIGURE 2:**
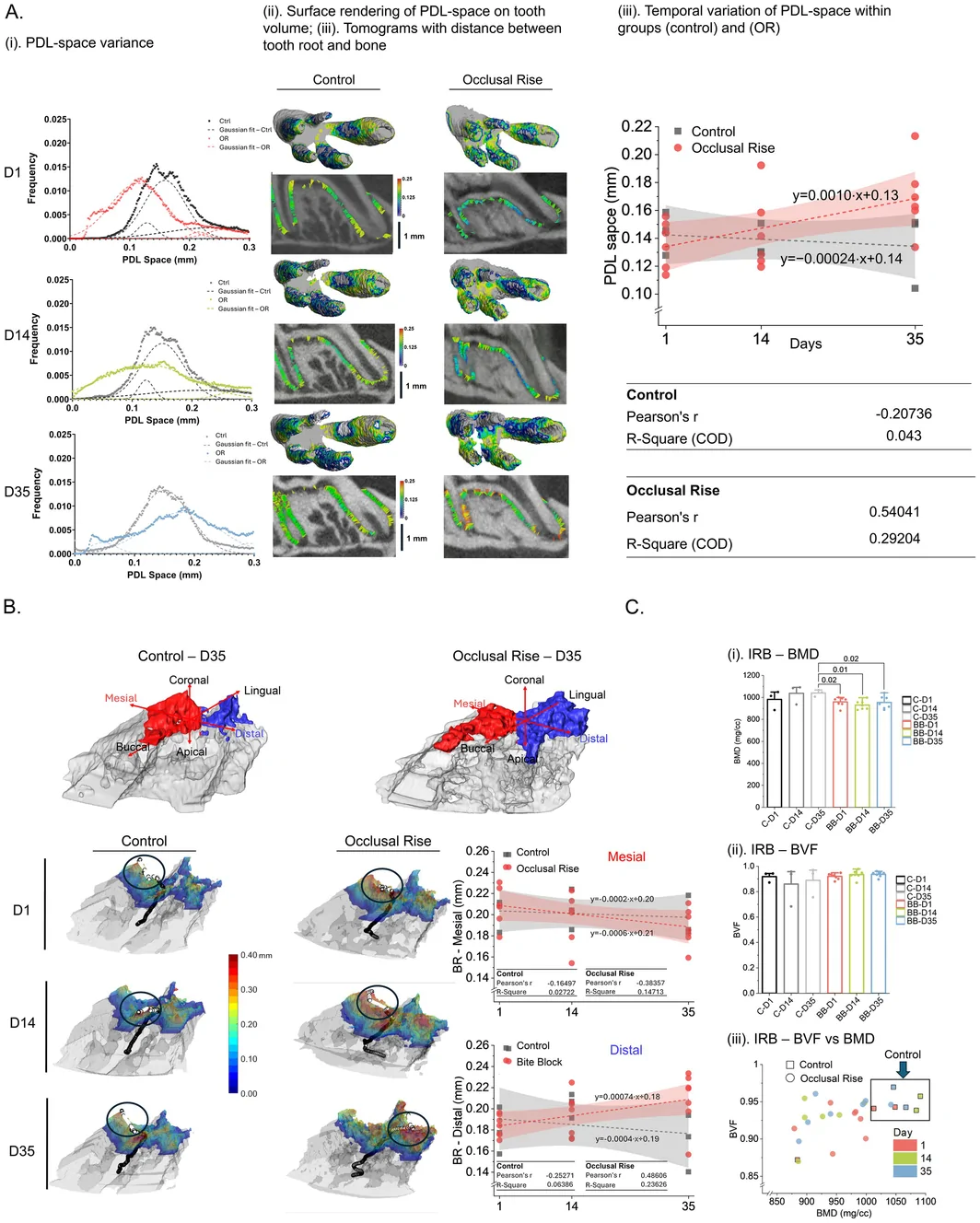
Widened PDL‐space, increased interradicular bone roughness, decreased BMD, and increased BVF trends in rats with occlusal rise compared to controls. (A) PDL‐space of the first left molar in the occlusal rise and control groups for D = 1,14, and 35 days are shown as a colour map on the tooth surface. A Gaussian fit is applied to the graphed PDL‐spaces with frequency of occurrence. Orthogonal slices of the tooth‐root interface were overlaid with calculated PDL‐space (surface distance vectors bounded to +/− 1 SD). The control group showed a slightly gradual decrease in PDL‐space, while PDL‐space in rats in the OR group was wider. (B) Topography of 0.4 mm depth interradicular bone surface is shown as mesial (red) and distal (blue) sides. Cumulative measurements of coronal‐apical vertical height were quantified to evaluate remodelling (formation and resorption) of the interradicular bone surface. Temporal plots of shifts in the centroid of the IRB illustrated a significant overlap between the two groups, indicating no difference in the mesial side compared to a divergence in data from D15 through D35 on the distal side of the IRB. (C) Significant differences in BMD of the IRM in the OR group at all time points relative to D35 of the control were observed (*p* < 0.02) (i). No differences in BVF between the two groups at all time points were observed (ii). Correlating BVF to BMD clustered data points from controls (black box with a block arrow) with a spread of data from the OR group (including lower BMDs and BVFs relative to the control) (iii).

Figure 2B depicts the temporal trends in bone surface roughness at the mesial and distal regions in both the OR and control groups, along with their respective linear regression results. Mesial and distal regions were chosen as the regions of examination because of the natural distal drift of the rat molars, which under normal conditions (chewing on HF only) encourages compression‐induced bone resorption in the distal side and tension‐induced bone formation on the mesial side. In the mesial region, the OR group exhibited a slight decreasing trend in surface roughness (y = 0.0002·x + 0.20), whereas the control group showed a more pronounced decline (y = −0.0006·x + 0.21). This suggests that sustained functional loading may induce localised remodelling of the bone and consequently a change in surface microarchitecture.

In the OR group, distal surface roughness increased significantly over time (y = 0.0007·x + 0.18), while the control group remained relatively stable or exhibited a slight decrease (y = −0.0004·x + 0.19). These findings indicate that the distal region is more susceptible to microstructural perturbations under aberrant mechanical loading, alluding to a dominance of resorption of mineral over formation of mineral, a nonuniform PDL‐space that could alter DAJ biomechanics. Collectively, the time‐dependent variations in bone surface roughness reveal region‐specific sensitivities to changes in the mechanical environment. In particular, the distal region appears more responsive to prolonged OR‐induced stimulation, potentially reflecting its critical role in stress transmission and bone remodelling processes.

Figure 2C presents BMD (i), BVF (ii), and a correlation between bone mineral density (BMD) and bone volume fraction (BVF) of the IRB (iii). Scatter plot analysis revealed a positive correlation between BVF and BMD (iii), indicating a coordinated relationship between bone quantity and mineralization during the remodelling process. Most data points from the OR group clustered in the lower region of the graph, reflecting degenerative changes in both the structural and compositional aspects of the bone. Importantly, the OR group showed markedly increased inter‐individual variability at D35, which may indicate high heterogeneity in bone remodelling responses under aberrant loads. These findings indicate that prolonged OR can significantly impact the morphology of the periodontal complex and could potentially impair its normal buffering and stress‐transmission functions.

### 3.3 Condyle Adaptation

Figure 3A illustrates the three‐dimensional reconstruction of trabecular bone porosity and the associated pore diameter distribution trends within the condylar cancellous bone at D1, D14, and D35 in both the OR and control groups. The colour‐coded 3D reconstructions visualise local pore diameter variations, highlighting the structural complexity and spatial heterogeneity of the internal porosity within the condyle (Figure 3C).

**FIGURE 3:**
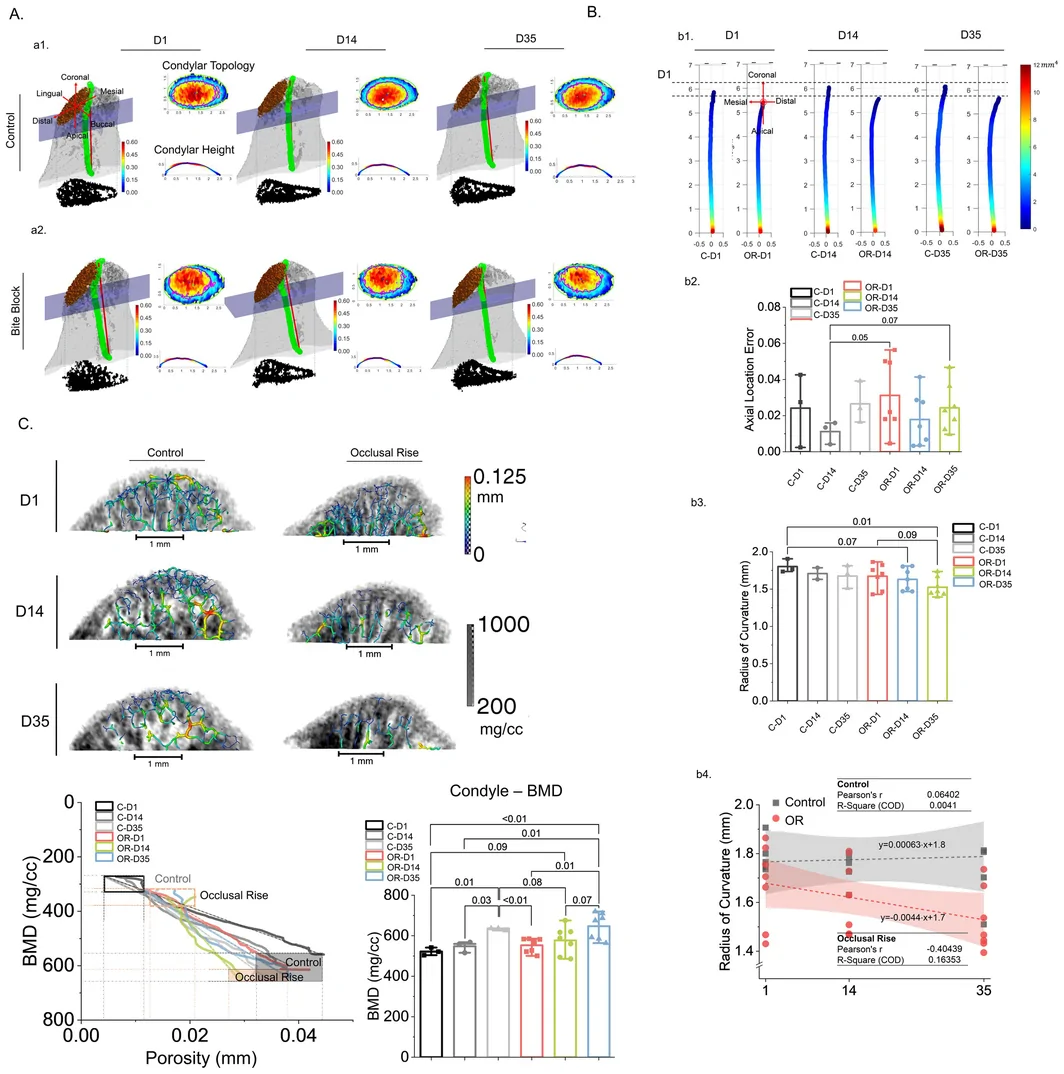
Chronic aberrant loading induced by the occlusal rise produced spatiotemporal alterations in TMJ condylar topology and bone morphology. Further, the association between BMD and porosity is steeper in the occlusal rise group compared to the age‐matched control group. (A) Condylar surface topology (surface height variation) at the condyle–fossa contact region is shown at Days 1 (D1; 12 weeks), 14 (D14; 14 weeks), and 35 (D35; 17 weeks). Condylar height differences within the top 0.4 mm of the contact surface are colour‐coded from blue to red (see scale bar). Cross‐sections taken at one‐third of the condylar stem length (purple planes) are shown across time points. The anatomical axis, represented by the centroidal line (green), and the mechanical axis, defined by a line connecting the proximal and distal ends of the anatomical axis (red), are illustrated for control (a1) and OR groups (a2). (B) (b1) Polar moment of inertia values are mapped by colour for each condylar layer. (b2) Deviation between the anatomical and mechanical axes reflects anatomy‐specific morphological adaptations over time at D1, D14, and D35 in both groups. At D14, both groups demonstrated reduced deviation and closer alignment between anatomical and mechanical axes, suggesting a transient adaptive response. The bar chart illustrates decreasing trends toward increased significance in the average distance between anatomical and mechanical axes over time (*p* < 0.1). (b3) Bar plots demonstrate decreasing trends in condylar radii of curvature with increasing statistical significance over time (*p* < 0.1). (b4) Linear regression models with 95% confidence intervals illustrate group‐specific trends in the radius of curvature within the condylar contact region. (C) Depth profile association across time points between BMD and porosity highlight temporal and group‐specific shifts in trabecular architecture. Profiles from the top 20% of the condylar trabecular region are highlighted (red box) and shown with linear fits and corresponding 95% confidence intervals (grey shading).

In the control group, the pores appeared relatively compact and regularly arranged, with a smooth gradient of pore diameters, indicating the preservation of a well‐organised trabecular network under normal occlusal loading (Figure 3B). In contrast, the OR group exhibited progressively sparser and disorganised pore structures, with notably enlarged channels evident by day 35, suggesting substantial internal bone remodelling driven by sustained abnormal mechanical stimuli. The control group displayed relatively stable curves, indicative of high structural homogeneity. In contrast, the OR group showed steeper slopes and pronounced temporal differences, reflecting a clear time‐dependent expansion of pores and increased heterogeneity in trabecular architecture under hyper‐occlusal loading (Figure 3B). These results indicate that abnormal functional loading not only drives structural remodeling but also significantly disrupts mineralization in the condylar bone.

**FIGURE 4:**
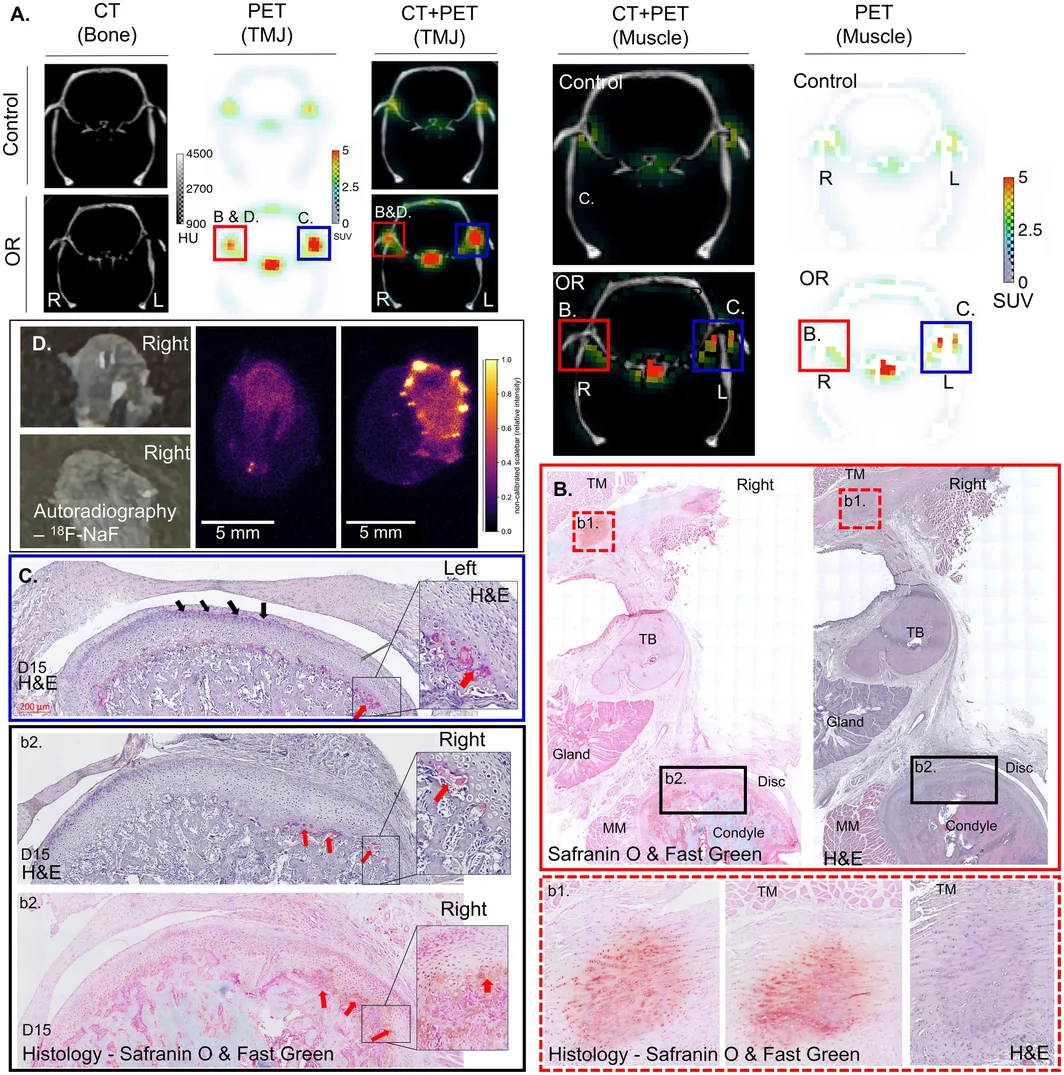
Multimodal imaging reveals TMJ responses to aberrant loading in the occlusal rise (OR) model. (A) Multimodal X‐ray CT and positron emission tomography (PET) imaging with ^18^F‐NaF of control rat heads and heads of rats subjected to OR with regions containing TMJs (red and blue boxes) are shown. PET SUV intensity reflects tissue metabolic activity and may arise from bone as well as adjacent fibrous tissues, particularly under conditions of mineralization or elevated cellular activity. In the OR model, the left TMJ at day 15 (D15) exhibited a PET‐positive signal that trended higher than controls. Both right (R) and left (L) condyles were affected, indicating a bilateral response to eccentric occlusal loading. (B) Histological examination using Safranin Orange (Safranin O) with fast green counterstain revealed ectopic cartilage‐like tissue transformation within adjacent softer tissues (b1, red‐dashed box). Furthermore, increased cellular activity was observed at the subchondral‐trabecular bone interface (red block arrows) in both Safranin O/fast green and H&E. (C) The left condyle was more uniformly affected and demonstrated increased cellular activity at the cartilage–trabecular bone interface (red block arrows), along with eosin‐positive metabolically active cells (black solid arrows) within the proliferative zone between fibrous and hypertrophic cartilage layers in both left and right condyles. (D) ^18^F‐NaF autoradiography revealed PET‐positive signal within cartilage and cortical bone of the right condyle, suggesting adaptive remodelling in response to OR–induced loading on the left DAJ. Abbreviations: TM, temporalis muscle; MM, masticatory muscle; TB, temporal bone; D15, day 15; CT, computed tomography; PET, positron emission tomography; SUV, standard uptake value.

### 3.4 Kinetic ^18^F‐NaF Standard Uptake Values in DAJ and TMJ

In the OR model, the left TMJ at day 15 (D15) exhibited a PET‐positive signal that trended higher than control (Figure 4A). The increase in SUV in the OR model in the right and left condyles (red box **B** and blue box **C**) compared to the control group indicated bilateral response to eccentric occlusal loading. Autoradiography confirmed detectable PET signal in the right TMJ and revealed PET‐positive signal within cartilage and cortical bone (Figure 4D), suggesting adaptive remodelling in response to occlusal rise–induced loading. Histological examination using Safranin O with Fast Green counterstain revealed ectopic cartilage‐like tissue transformation within adjacent softer tissues (red dashed box) in agreement with the PET signal corresponding to the right TMJ soft‐tissue region (Figure 4A, right panel, red box B). In contrast, the left condyle (Figure 4A, blue box C) was more uniformly affected, confirming the higher PET‐positive signal within both osseous and adjacent soft tissues (Figure 4A, blue box C, right panel; adjacent softer tissues are also PET positive). Increased cellular activity was observed at the cartilage–trabecular bone interface (block red arrows, left‐top panel), along with eosin‐positive metabolically active cells (black solid arrows) within the proliferative zone between fibrous and hypertrophic cartilage layers in both left and right condyles (Figure 4B,C).

### 3.5 Coupled Biomechanical Analysis of the Alveolar Bone and Temporomandibular Joint Under Functional Loading

Temporal evolution of von Mises stress distribution in the mandible, condyle, and first molar regions under occlusal loading is shown in Figure 5B (b2 and b3). In the control group, the condylar apex and posterior margin consistently exhibited stress concentration, with the stress distribution remaining spatially stable over time. In contrast, the OR group displayed a marked reduction in stress during the first two weeks, suggesting an initial phase of mechanical adaptation. However, stress levels progressively increased thereafter, indicating that prolonged OR stimulation led to compromised structural stability and progressive accumulation of stress in the joint region.

**FIGURE 5:**
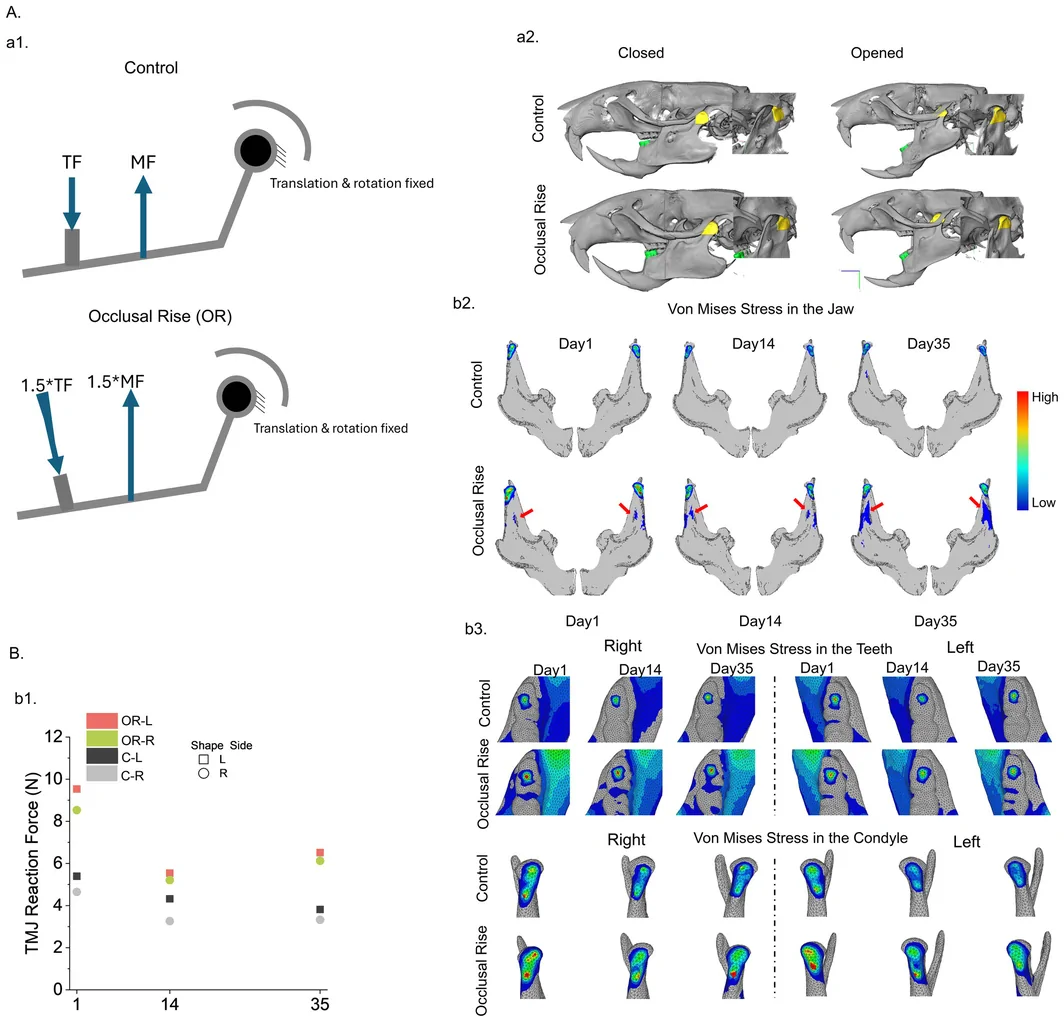
Finite element analysis of CT‐derived jaw models demonstrates the relationship between occlusal loading and TMJ forces over time. Finite element analysis was performed on CT scans of adapted jaws at Days 1 (D1), 14 (D14), and 35 (D35) to evaluate the association between occlusal forces and mechanical loading of the temporomandibular joint (TMJ). (A) (a1) Link diagram illustrates boundary conditions. In the occlusal rise (OR) model, both tooth force (TF) and muscle force (MF) were set at 1.5‐fold higher than in controls. Reaction forces at the TMJ were calculated as the forces required to prevent translational and rotational motion in response to applied occlusal and muscle forces. (a2) Three‐dimensional renderings highlighting the TMJ condyle and the molar receiving the OR under closed‐ and open‐mouth conditions. (B) (b1) Reaction forces at the left (squares) and right (circles) TMJs in control (black and grey) and OR (red and green) groups at D1, D14, and D35. The OR group exhibited higher initial TMJ forces at D1, followed by a reduction at D14 and a modest increase at D35 relative to controls. The difference between left and right TMJ forces progressively converged over time, indicating mechanical adaptation. (b2) Von Mises stress distributions mapped onto CT‐derived models of control (top row) and OR (bottom row) jaws at D1, D14, and D35. (b3) Quantification of von Mises stresses in the teeth and corresponding TMJs demonstrates elevated TMJ stresses immediately following occlusal loading at D1, a reduction at D14, and a subsequent increase at D35, with greater stress observed on the right condyle compared to the left.

Meanwhile, Figure 5B (b4) reveals an asymmetric stress distribution between the left and right condyles, suggesting differences in bilateral load transmission pathways under occlusal loading. This asymmetry may reflect unequal joint loading or adaptive morphological changes in response to altered functional demands. It also demonstrates that stress in the interradicular region of the first molar is primarily concentrated in the area between the mesial and distal roots. In the OR group, this stress concentration is markedly increased compared to the control group, indicating a forward shift in occlusal force distribution toward the anterior dentition.

## 4 Discussion

Disruption of occlusal harmony through increased vertical dimension, malocclusion, sustained mouth opening, or OR has been linked to pathological remodelling of both the DAJ and the TMJ, often culminating in temporomandibular disorders (TMD) and impaired mandibular development [15, 24, 25, 26]. Sustained OR produces premature or excessive tooth contacts that alter mandibular biomechanics, leading to reductions in alveolar bone height, decreased masseter muscle mass, and changes in bone volume and cortical thickness [9, 11, 15, 27, 28, 29]. These biomechanical perturbations tune the distribution of mechanical loads, reflecting an intrinsic optimisation mechanism essential for the development and preservation of dentofacial architecture.

In the present study, OR was employed as a model of sustained non‐physiological mechanical stimulation, exerting significant effects on both DAJ and TMJ structures (Figures 2 and 3). Longitudinal micro‐CT analysis revealed widened PDL space in the DAJ, accompanied by spatially specific bone resorption and formation within the interradicular region (Figure 2A,B). Bone mineral density (BMD) was reduced across all evaluated time points in the OR group, while bone volume fraction (BV/TV) remained relatively preserved, indicating redistribution rather than uniform loss of mineralised tissue (Figure 2C). These findings suggest that the interradicular bone in the DAJ achieves local mechanical equilibrium through modulation of bone remodelling rates and directional adaptation from mesial to distal (Figure 2B). While bidirectional remodelling has been demonstrated in orthodontic tooth movement models, the present work also confirms its existence in a vertically overloaded system (Figure 2A) but without mesial or distal movement of teeth as is done in orthodontic intervention or experimental tooth movement [30, 31, 32], expanding current understanding of load‐regulated remodelling of the interradicular bone (Figure 2).

TMJ responses to occlusal rise exhibited systemic and temporally staged characteristics. Morphometric analysis revealed progressive alterations in the width between the condyles, curvature, and surface topology over time (Figures 1B and 3A–B), with associated changes in trabecular spacing and internal bone architecture (Figure 3C). During early phases following OR, redistribution of subchondral BMD and changes in condylar geometry could suggest an “underloading” or stress‐shielding adjustment strategy aimed [33, 34] at protecting bone, fibrocartilage, and articular cartilage tissues. Alternatively, these changes may indicate that loading has exceeded tissue tolerance thresholds, initiating inflammation‐associated degenerative processes [27, 35, 36]. With continued loading, recovery of mechanical stability appeared to be mediated by increased bone density and altered load‐transmission pathways, consistent with nonlinear adaptive regulation at the bone–cartilage interface [35, 36].

Multimodal PET/CT imaging [37] further revealed that occlusal rise induces metabolic activation not only in the bony compartments but also within adjacent fibrous tissues, including ligament, muscle, and tendon (Figure 4A,B). PET‐positive signals were observed bilaterally despite eccentric loading, indicating a coordinated TMJ response rather than a purely localised adaptation, confirming that both working and balancing sides can undergo mechanoadaptation. Autoradiography and histology confirmed metabolically active cartilage, cortical bone remodelling, and ectopic cartilage‐like tissue formation within soft‐tissue regions (Figure 4C,D). These findings align with prior rodent studies demonstrating that mechanical loading upregulates chondrogenic markers (e.g., collagen II, aggrecan, Sox9) under moderate loads, while sustained or excessive loading increases osteoclastogenesis and RANKL expression, shifting the balance toward catabolic remodelling not limited to the TMJ but can also be extended to the DAJ (Figures 4 and S4) [27, 35].

Despite several limitations, finite element analyses provided a mechanical framework to link these observations to the evolving load environments (Figures 5 and S5). Occlusal rise produced elevated TMJ reaction forces immediately following intervention, a finding that is inconsistent with a primary stress‐shielding mechanism and instead supports a stress‐threshold‐based response. Over time, TMJ stresses decreased (D14) and subsequently increased (D35), indicating a temporally staged mechanical adaptation (Figure 5B). Von Mises stress mapping revealed a characteristic “buffering‐then‐restructuring” pattern (from D1–14 could be buffering, and D14–D35 restructuring), whereby early stress attenuation is followed by structural reorganisation and renewed load bearing (Figure 5B,C). This observation raises the question of whether the structural and biological changes in the DAJ and TMJ are reversible upon the removal of occlusal rise within a two‐week window. This coupling between multiscale DAJ and TMJ parameters supports the existence of a coordinated biomechanical regulatory network rather than isolated compartmental responses.

Through occlusal rise, adaptive responses are generated not only in the DAJ but also in the TMJ, perpetuated primarily by eccentric loading (Supporting Information for Clinical Scenarios). Collectively, integration of topic‐relevant literature [7, 9, 10, 11, 18, 28, 35, 36, 38, 39, 40] with our multiscale imaging, morphometric, and computational analyses demonstrates that occlusal rise–induced eccentric loading drives several responses spanning early metabolic activation, structural adaptation, and longer‐term remodelling of both the DAJ and TMJ. Identification of sustained forces from occlusal rise as a central driver of these changes provides a biologically grounded basis for translating animal model findings into clinical strategies (see Supporting Information) aimed at diagnosing, monitoring, and treating occlusion‐related temporomandibular disorders.

## 5 Limitations

Limitations in this study are as follows:

- Forces induced by the OR are a controlled yet simplified form of malocclusion; it doesn't represent the full spectrum or complexity of clinical malocclusion.
- In the FEA model, the whole jaw was considered as a single unit. However, the properties should be calibrated by the BMD and at least divided into tooth, cortical, and trabecular bone [41].
- The condyles were fixed to simulate the attached ligament, but with no disc between the condyle and fossa (disc provides frictionless or low‐friction sliding constraint, which does not exist in this model). Fixing the degrees of freedom of the condyles could cause stress concentration at the fixed nodes, near the condyles.
- The muscular force was simplified to only masseter force, ignoring other influential muscles including the pterygoids and their connections between the maxillary and mandibular jaws [42, 43, 44].
- Simulations at different masseter muscle forces, possibly 2, 5, and 10 times more than the occlusal force, can help understand the combined effect of masseter and occlusal force on the TMJ [43, 44].
- Force directions were also simplified to an angular motion, contrasting a real situation which involves translation and rotation, allowing an elliptical pattern of the jaw during chewing [41, 44].
- Overall, this FEA model is only used to examine the effect of the shape factor caused by the OR for conceptual validation.
- Although structural, metabolic, and mechanical changes were quantified, the molecular pathways mediating these responses are yet to be assessed.
- Outcomes such as pain and its association with masticatory efficiency are also further warranted.

## Author Contributions

**Robert Flavell:** formal analysis. **Zhiyuan Yang:** investigation. **Brandon Lee:** investigation. **Haochen Ci:** formal analysis, methodology, investigation, writing – original draft, visualization. **Youngho Seo:** formal analysis. **Umama Ali:** methodology, investigation, formal analysis, writing – original draft, visualization. **Alireza Hatamifar:** writing – original draft. **Nhu Huynh:** writing – original draft. **Rishi J. Gupta:** validation. **Bo Wang:** formal analysis. **Yongmei Wang:** investigation. **Rebeka Silva:** validation. **Ayush Bakhandi:** formal analysis, visualization. **Christine Nguyen:** formal analysis. **Sunita P. Ho:** writing – original draft, reviewing and editing, formal analysis, conceptualization, supervision, funding acquisition.

## Funding

This work was supported by NIDCR R56DE036307(SPH).

## Conflicts of Interest

The authors declare no conflicts of interest.

## Supporting information


**Figure S1:** (A) Daily weight of rats up to 35 days, with weights at D1, D14, and D35, are shown. (B) Representative micro‐CT images showing the occlusal surface morphology of the bite block and the occlusal relationship between the maxillary and mandibular molars (in yellow). (C) Bone roughness values of both the control and OR groups were higher at the distal regions compared to the mesial regions. In addition, in the OR group at D14, the bone roughness values on the mesial and distal regions were similar. (D) Surface topographies of the left and right condyles in control and occlusal rise groups are shown.
**Figure S2:** Bone mineral density and polar moment of inertia estimates. (A) Calibration phantom and the resulting calibration curve obtained for the micro‐CT scanner. (B) Schematic illustrating calculation of the polar moment of inertia (Ip) of the TMJ condylar bone. Ip is computed for a differential area (dA) located at coordinates dx and dy. The Ip‐values for multiple such regions within the condylar cross‐section are calculated and summed (Σ) to obtain the Ip for a given condylar section. Ip was evaluated across multiple sections of the condyle, and these values are plotted and shown in Figure 3.
**Figure S3:** (A) Porosity and BMD of the condyles: (a1) The volume (colour map) and depth (average of porosities at each depth) profiles of porosities from the condylar surface into the trabecular space are shown. The porosity in the first 300 μm, representative of the condylar subsurface, is shown within the red box. At D1, the profiles are similar between control and OR; however, at days 14 and 35, the profiles were different from those observed in controls and contained smaller‐diameter pores. (a2) The bone mineral density (BMD) profiles indicated a BMD increase with depth and a decrease in porosity in the OR compared to age‐matched control groups. The first 300 μm of depth are shown in the red box.
**Figure S4:** PET scans of the DAJs in control and occlusal rise (OR) groups are shown. (A) Half‐body including head with correlated signals from PET and CT that could include signals from bone and muscle, PET only from bone and muscle, and PET only from muscle (after subtracting the PET signal from bone). (B) Higher magnification of the head only with DAJ highlighted as regions C, and corresponding PET and CT scans. (C) At 15 min, the standard uptake value from the PET scan of the OR group under open mouth conditions was greater than control, which subsequently increased over time (60 min). (D) The 18F‐NaF uptake is shown for the condyles from control and OR groups. Note: The ectopic tissue transformation in the softer tissues adjacent to the condyle from the affected masticatory complex.
**Figure S5:** Link‐based biomechanical model illustrates eccentric loading. (A) The anatomical axis was defined as the midsagittal plane of the skull (yellow dashed line). (B) In the occlusal rise (OR) group, the mechanical axis is oblique in the transverse plane because the OR alters load transmission pathways when the jaws are considered as an integrated system of the masticatory complex. The OR on the working side acts as a fulcrum during mastication, and transforms the mandible into a lever, resulting in a greater torque (M) by the right condyle on the balancing side. Consequently, during attempted jaw closure, both the muscular forces (Fm) and the corresponding temporomandibular joint (TMJ) reaction forces (FRe) are increased bilaterally.

## Data Availability

The data that support the findings of this study are available from the corresponding author upon reasonable request.
