# Supplementary material for: Eccentric Occlusal Loads Remodel the Dentoalveolar and Temporomandibular Biomechanical Unit in Rats
Source: J Oral Rehabil. 2026 Aug 17;53(12):2366–78. doi: 10.1111/joor.70284 (PMC13755653; doi:10.1111/joor.70284)
Supplement: Supplementary file 1 [file JOOR-53-2366-s001.docx]

**Eccentric Occlusal Loads Remodel the Dentoalveolar and Temporomandibular Biomechanical Unit in Rats**

Haochen Ci^1, 2, #^, Umama Ali^3, #^, Zhiyuan Yang^1, 4^, Ayush Bakandi^1^, Nhu Huynh^1^, Christine Nguyen^1^, Brandon Lee^1, 5^, Robin Peter^3,7^, Youngho Seo^3,8,9^, Robert Flavell^3,9,10^, Alireza Hatamifar^6^ , Rishi J. Gupta^11^, Rebeka Silva^11^, Yongmei Wang^1^, Bo Wang^2^, Sunita P. Ho^1,*^

^1^Department of Preventive and Restorative Dental Sciences, School of Dentistry, University of California San Francisco; ^2^International Research Center for Computational Mechanics, Dalian University of Technology, Dalian 116023, P.R. China; ^3^Department of Radiology and Biomedical Imaging, School of Medicine, University of California San Francisco, San Francisco, CA; ^4^School of Dentistry, University of Washington, Seattle, WA; ^5^Neuroscience Graduate Program, Department of Pharmacology, University of California, Davis, Davis, CA; ^6^Department of Oral and Maxillofacial Surgery, University of California San Francisco, ^7^Department of Radiation Oncology, School of Medicine, University of California San Francisco, San Francisco, CA, ^8^Department of Nuclear Engineering, University of California, Berkeley, CA USA, ^9^UCSF Helen Diller Family Comprehensive Cancer Center, San Francisco, CA-94107, USA, ^10^Department of Pharmaceutical Chemistry, University of California, San Francisco, CA USA, ^11^Department of Oral and Maxillofacial Surgery, VA Medical Center, San Francisco, CA, USA

^#^ Equal contribution to this study

* To whom correspondence should be addressed:

Sunita P. Ho, Ph.D.

513 Parnassus Avenue, HSW 813

University of California San Francisco

San Francisco, CA 94143

Phone: 415-514-2818

**Keywords:** dentoalveolar joint, temporomandibular joint, masticatory complex, occlusal loads, biomechanics

**Clinical Relevance of the Animal Model:** Over 5-12% of the population is affected by temporomandibular disorders (TMDs) (1, 2). Temporomandibular joint osteoarthritis (TMJOA), a **TMD subtype** is a degenerative joint disease (DJD) which affects TMJ biomechanics and joint function (3, 4). TMJOA is characterized by progressive degeneration of cartilage, subchondral bone, and associated joint structures, with radiographic and structural alterations commonly observed in affected patients (5, 6). Despite extensive characterization of clinical symptoms and imaging features, the initiating biomechanical and biological events that precipitate progressive joint degeneration remain poorly defined. Elucidating how altered occlusal mechanics drive coordinated adaptations across the DAJ and TMJ is therefore critical for advancing mechanistically informed prevention and treatment strategies. Several clinically relevant applications of these mechanistic insights are discussed below.

**1)	Bruxism as a Driver of TMJ Degeneration**

Bruxism is a common parafunctional habit characterized by repetitive jaw clenching, tooth grinding, and lateral excursive movements, and represents a clinically relevant source of excessive mechanical loading to both the TMJ and DAJ. These abnormal forces can damage cartilaginous tissues, osseous structures, and the subchondral bone–cartilage interface (7-9). Bruxism occurs during both wakefulness and sleep; awake bruxism is more prevalent in females, whereas sleep bruxism demonstrates no gender bias (10). Notably, electromyography and bite force studies show that forces generated during sleep bruxism can exceed those produced during daytime activity, exposing the TMJ to substantial nocturnal mechanical insult. These excessive and repetitive loads are thought to initiate a self-perpetuating cycle of chronic mechanical stress that promotes progressive joint degeneration and increases susceptibility to osteoarthritis (5). Anatomically specific wear patterns observed across donor joints, including lateral wear associated with grinding and vertical damage linked to clenching, provide biomechanical evidence implicating bruxism in TMJOA pathogenesis (1, 5). Clinically, TMJOA manifests with articular joint and masticatory muscle pain, restricted mandibular motion, and impaired quality of life (11-13).

In the setting of chronic bruxism and sustained eccentric loading, myofascial pain involving the masticatory muscles commonly arises as part of a protective muscle-guarding response to increased joint loading. Elevated tonic and phasic muscle activity serves to stabilize the mandible, limit excessive joint motion, and redistribute occlusal forces in an effort to reduce articular stress. While this compensatory muscle guarding may initially protect joint structures, persistent activation leads to muscle fatigue, ischemia, and pain, contributing to the myofascial component of temporomandibular disorders.

Clinical interventions that target myofascial symptoms in isolation, such as chemodenervation of the masticatory muscles, do not address the underlying mechanical drivers of disease and may disrupt this compensatory stabilization. In the absence of concurrent management of parafunctional behavior or occlusal loading, attenuation of muscular force generation may alter load transmission, reduce joint protection, and potentially exacerbate articular degeneration. These considerations underscore the limitations of symptom-based treatment strategies and highlight the need for mechanistically informed interventions that address both muscular manifestations and the underlying biomechanical environment. Although TMD represents a major source of musculoskeletal pain (14, 15), current clinical approaches largely provide transient symptom relief without preventing disease progression (3, 16).

**2)	Implications for Implant-Supported and Fixed Prosthodontic Rehabilitation**

Altered joint mechanics also arise in the setting of implant-supported restorations and fixed partial dentures, where the adaptive capacity of the dentoalveolar joint may be reduced or absent. Unlike natural teeth, osseointegrated implants lack a periodontal ligament, thereby eliminating viscoelastic load buffering and mechanosensory feedback that normally support adaptive force redistribution within the dentoalveolar complex (17-20). Consequently, occlusal discrepancies or sustained eccentric loading associated with rigid prosthodontic restorations are more likely to be transmitted directly to the temporomandibular joint (21, 22).

The present findings indicate that when adaptive remodeling within the dentoalveolar joint is limited, mechanical compensation may be shifted toward the TMJ, increasing joint-level stress concentrations and promoting cartilage remodeling and subchondral bone reorganization. Under conditions of altered occlusion or chronic overload, patients with implant-supported or rigid fixed prostheses may therefore be at greater risk for accelerated or more severe TMJ structural changes (23, 24). These observations emphasize the need for careful occlusal design, load management, and longitudinal TMJ assessment in prosthodontic treatment planning, particularly in patients with parafunctional habits or pre-existing joint symptoms.

**3)	Relevance to Orthodontic Treatment and Orthognathic Surgical Planning**

Changes in occlusal relationships are inherent to both orthodontic treatment and orthognathic surgical workflows, where intentional modifications to dental alignment and jaw position alter TMJ loading (25, 26). During non-surgical orthodontic treatment, occlusal interferences, bite opening, and asymmetric force application can introduce eccentric loading patterns comparable to those modeled in this study (25, 27, 28). Similarly, during the pre-surgical orthodontic phase of orthognathic surgery, occlusal decompensation may transiently increase joint loading prior to definitive skeletal correction (21).

These findings underscore the importance of recognizing the TMJ as a load-responsive joint that undergoes time-dependent biological and mechanical adaptation in response to sustained occlusal perturbations. Systematic monitoring of TMJ morphology, symptoms, and function throughout orthodontic and pre-surgical phases may be critical for identifying patients at risk of maladaptive remodeling or disease progression (29). Integrating TMJ-focused assessment into orthodontic and surgical planning may help identify critical windows for intervention, reduce long-term joint sequelae, and improve functional outcomes.

**Conclusion**: Across these clinical contexts, the occlusal rise (OR) model provides mechanistic insight into how sustained alterations in occlusal mechanics drive coordinated, time-dependent adaptations across the dentoalveolar and temporomandibular joint. Chronic eccentric loading, whether arising from parafunction, reduced dentoalveolar adaptability following rigid prosthodontic rehabilitation, or intentionally altered occlusion during orthodontic and orthognathic treatment, emerges as a key determinant of joint-level biological and mechanical response. By defining tissue-specific and temporal responses to sustained occlusal perturbation, this model bridges experimental biomechanics with clinical TMJOA and offers a framework for identifying at-risk patients and critical windows for intervention to prevent irreversible joint dysfunction.

**Sample size determination for macroscale loading and microscale biochemical and physicochemical properties:** Based on previous data on elastic modulus values of cementum and dentin in rats (30), an estimate of the mean square error was obtained for: within locations (coronal to apical), locations within teeth (cementum to dentin), and teeth within groups that included those subjected to harder and softer foods in addition to with and without occlusal rise. Then, the differences among 4 groups (and among 5 times) ranging from 0.66 to 0.87 GPa (hardness ranges for bone and cementum) were examined (31).  With n=6 per group, a two-way analysis of variance (ANOVA) with a common standard deviation of 0.23 GPa in elastic modulus among groups is estimated to be detected with 80% power for group (variance of 0.006) and 80% power for time (variance of 0.006) using a 0.05-level F statistic.  Additionally, differences between normal specimens and specimens affected by load that are as large as 1.0 GPa will have greater power to detect statistical significance. Including the control group in the ANOVA will further increase power. We will use n = 7 per group in case of unexpected loss of a subject.

**Supplemental Figure 1. A.** Daily weight of rats up to 35 days with weights at D1, D14, and D35 are shown. **B.** Representative micro-CT images showing the occlusal surface morphology of the bite block and the occlusal relationship between the maxillary and mandibular molars (in yellow). **C.** Bone roughness values of both the control and OR groups were higher at the distal regions compared to the mesial regions. In addition, in the OR group at D14, the bone roughness values on the mesial and distal regions were similar. **D.** Surface topographies of the left and right condyles in control and occlusal rise groups are shown.

**Supplemental Figure 2. Bone mineral density and polar moment of inertia estimates. A.** Calibration phantom and the resulting calibration curve obtained for the micro-CT scanner. **B.** Schematic illustrating calculation of the polar moment of inertia (Ip) of the TMJ condylar bone. Ip is computed for a differential area (dA) located at coordinates *dx* and *dy*. The Ip values for multiple such regions within the condylar cross-section are calculated and summed (Σ) to obtain the Ip for a given condylar section. Ip was evaluated across multiple sections of the condyle, and these values are plotted and shown in Figure 3.

**Supplemental Figure 3. A. Porosity and BMD of the condyles: a1.** The volume (color map) and depth (average of porosities at each depth) profiles of porosities from the condylar surface into the trabecular space are shown. The porosity in the first 300 microns representative of the condylar subsurface is shown within the red box. At D1, the profiles are similar between control and OR, however, at days 14 and 35, the profiles were different from those observed in controls and contained smaller diameter pores. **a2.** The bone mineral density (BMD) profiles indicated a BMD increase with depth and a decrease in porosity in the OR compared to age-matched control groups. The first 300 microns of depth are shown in the red box.

**Supplemental Figure 4.** PET scans of the DAJs in control and occlusal rise (OR) groups are shown. **A.** Half-body including head with correlated signals from PET and CT that could include signals from bone and muscle, PET only from bone and muscle, and PET only from muscle only (after subtracting the PET signal from bone). **B.** Higher magnification of the head only with DAJ highlighted as regions C, and corresponding PET and CT scans. **C.** At 15 min, the standard uptake value from the PET scan of the OR group under open mouth conditions was greater than control, which subsequently increased over time (60 min). **D.** The ^18^F-NaF uptake is shown for the condyles from control and OR groups. Note: the ectopic tissue transformation in the softer tissues adjacent to the condyle from the affected masticatory complex.

**Supplemental Figure 5. Link-based biomechanical model illustrates eccentric loading. A.** The anatomical axis was defined as the midsagittal plane of the skull (yellow dashed line). **B.** In the occlusal rise (OR) group, the mechanical axis is oblique in the transverse plane because the OR alters load transmission pathways when the jaws are considered as an integrated system of the masticatory complex. The OR on the working side acts as a fulcrum during mastication, and transforms the mandible into a lever, resulting in a greater torque (*M*) by the right condyle on the balancing side. Consequently, during attempted jaw closure, both the muscular forces (F_m_) and the corresponding temporomandibular joint (TMJ) reaction forces (F_Re_) are increased bilaterally.

**REFERENCES**

1.	Bianchi J, Ruellas ACD, Gonçalves JR, Paniagua B, Prieto JC, Styner M, et al. Osteoarthritis of the Temporomandibular Joint can be diagnosed earlier using biomarkers and machine learning. Scientific Reports. 2020;10(1).

2.	NIDCR. Prevalence of TMJD and its Signs and Symptoms: National Institute of Dental and Craniofacial Research; 2018 [

3.	Silva MAG, Pantoja LLQ, Dutra-Horstmann KL, Valladares-Neto J, Wolff FL, Porporatti AL, et al. Prevalence of degenerative disease in temporomandibular disorder patients with disc displacement: A systematic review and meta-analysis. J Craniomaxillofac Surg. 2020;48(10):942-55.

4.	Cardoneanu A, Macovei LA, Burlui AM, Mihai IR, Bratoiu I, Rezus, II, et al. Temporomandibular Joint Osteoarthritis: Pathogenic Mechanisms Involving the Cartilage and Subchondral Bone, and Potential Therapeutic Strategies for Joint Regeneration. Int J Mol Sci. 2022;24(1).

5.	Immonen J, Patterson D, Kent N, Pipkin S, Luu A, Nguyen LM, et al. Biomechanics of Bruxism Potentially Determine the Sites of Severe TMJ Osteoarthritis. Biomechanics-Basel. 2024;4(2):369-81.

6.	Reddy SV, Kumar MP, Sravanthi D, Mohsin AH, Anuhya V. Bruxism: a literature review. J Int Oral Health. 2014;6(6):105-9.

7.	Burr DB, Radin EL. Microfractures and microcracks in subchondral bone: are they relevant to osteoarthrosis? Rheum Dis Clin North Am. 2003;29(4):675-85.

8.	Burr DB. The importance of subchondral bone in osteoarthrosis. Curr Opin Rheumatol. 1998;10(3):256-62.

9.	Burr DB. Anatomy and physiology of the mineralized tissues: role in the pathogenesis of osteoarthrosis. Osteoarthritis Cartilage. 2004;12 Suppl A:S20-30.

10.	Nishigawa K, Bando E, Nakano M. Quantitative study of bite force during sleep associated bruxism. J. Oral Rehabil. 2001;28(5):485-91.

11.	Ohrbach R, Dworkin SF. The Evolution of TMD Diagnosis: Past, Present, Future. J Dent Res. 2016;95(10):1093-101.

12.	Pizzo PA, Clark NM. Relieving PAIN in America A Blueprint for Transforming Prevention, Care, Education, and Research Introduction. Relieving Pain in America: A Blueprint for Transforming Prevention, Care, Education, and Research. 2011:19-53.

13.	Steglitz J, Buscemi J, Ferguson MJ. The future of pain research, education, and treatment: a summary of the IOM report "Relieving pain in America: a blueprint for transforming prevention, care, education, and research". Transl Behav Med. 2012;2(1):6-8.

14.	Bagis B, Ayaz EA, Turgut S, Durkan R, Özcan M. Gender Difference in Prevalence of Signs and Symptoms of Temporomandibular Joint Disorders: A Retrospective Study on 243 Consecutive Patients. Int J Med Sci. 2012;9(7):539-44.

15.	Cooper BC, Kleinberg I. Examination of a large patient population for the presence of symptoms and signs of temporomandibular disorders. Cranio-the Journal of Craniomandibular & Sleep Practice. 2007;25(2):114-26.

16.	Melou C, Pellen-Mussi P, Jeanne S, Novella A, Tricot-Doleux S, Chauvel-Lebret D. Osteoarthritis of the Temporomandibular Joint: A Narrative Overview. Medicina (Kaunas). 2022;59(1).

17.	Song D, Shujaat S, Politis C, Orhan K, Jacobs R. Osseoperception following dental implant treatment: A systematic review. J Oral Rehabil. 2022;49(5):573-85.

18.	Sadowsky SJ, Brunski JB. Are teeth superior to implants? A mapping review. J Prosthet Dent. 2021;126(2):181-7.

19.	Abarca M, Van Steenberghe D, Malevez C, Jacobs R. The neurophysiology of osseointegrated oral implants. A clinically underestimated aspect. J Oral Rehabil. 2006;33(3):161-9.

20.	Chang HH, Yeh CL, Wang YL, Huang YC, Tsai SJ, Li YT, et al. Differences in the biomechanical behaviors of natural teeth and dental implants. Dent Mater. 2021;37(4):682-9.

21.	Zhu Y, Zheng F, Gong Y, Zhu J, Yin D, Liu Y. Effect of occlusal contact on TMJ loading during occlusion: An in silico study. Comput Biol Med. 2024;178:108725.

22.	Lin CL, Wang JC, Chang WJ. Biomechanical interactions in tooth-implant-supported fixed partial dentures with variations in the number of splinted teeth and connector type: a finite element analysis. Clin Oral Implants Res. 2008;19(1):107-17.

23.	Wu Z, Lin Y, Lin Y. Impact of age on degenerative joint disease of the temporomandibular joint: A systematic review and meta-analysis. Medicine (Baltimore). 2025;104(17):e41915.

24.	Murphy MK, MacBarb RF, Wong ME, Athanasiou KA. Temporomandibular disorders: a review of etiology, clinical management, and tissue engineering strategies. Int J Oral Maxillofac Implants. 2013;28(6):e393-414.

25.	Myllymaki E, Heikinheimo K, Suominen A, Evalahti M, Michelotti A, Svedstrom-Oristo AL, et al. Longitudinal trends in temporomandibular joint disorder symptoms, the impact of malocclusion and orthodontic treatment: A 20-year prospective study. J Oral Rehabil. 2023;50(9):739-45.

26.	Verhelst PJ, Van der Cruyssen F, De Laat A, Jacobs R, Politis C. The Biomechanical Effect of the Sagittal Split Ramus Osteotomy on the Temporomandibular Joint: Current Perspectives on the Remodeling Spectrum. Front Physiol. 2019;10:1021.

27.	Wang M, Wang J, Jin X, Liu D, Bian H, Zhao Y, et al. Impact of occlusal contact pattern on dental stability and oromandibular system after orthodontic tooth movement in rats. Sci Rep. 2023;13(1):22276.

28.	Gurbanov V, Bas B, Oz AA. Evaluation of Stresses on Temporomandibular Joint in the Use of Class II and III Orthodontic Elastics: A Three-Dimensional Finite Element Study. J Oral Maxillofac Surg. 2020;78(5):705-16.

29.	Szczesniak M, Issa J, Ciszewska A, Okla M, Galczynska-Rusin M, Dyszkiewicz-Konwinska M. Alterations in the Temporomandibular Joint Space Following Orthognathic Surgery Based on Cone Beam Computed Tomography: A Systematic Review. J Clin Med. 2025;14(20).

30.	Ho SP, Yu B, Yun W, Marshall GW, Ryder MI, Marshall SJ. Structure, chemical composition and mechanical properties of human and rat cementum and its interface with root dentin. Acta Biomater. 2009;5(2):707-18.

31.	Niver EL, Leong N, Greene J, Curtis D, Ryder MI, Ho SP. Reduced functional loads alter the physical characteristics of the bone-periodontal ligament-cementum complex. J Periodontal Res. 2011;46(6):730-41.
